# The innovation paradox in human-AI symbiosis: ambidextrous effects of AI technology adoption on innovative behavior

**DOI:** 10.3389/frai.2025.1635246

**Published:** 2025-10-21

**Authors:** Xin Wang, Lin Long

**Affiliations:** ^1^School of Trade Union Studies, China University of Labor Relations, Beijing, China; ^2^College of Management Science, Chengdu University of Technology, Chengdu, China

**Keywords:** AI technology adoption, job demands-resources model, task crafting, employee innovative behavior, felt obligation for constructive change

## Abstract

**Introduction:**

AI is radically changing workplace ecosystems in the midst of the Fourth Industrial Revolution, making human-machine collaboration a need for organizations. The ambidextrous processes by which AI simultaneously encourages and constrains inventive behaviors need systematic examination, even though employee innovation is still essential for maintaining competitive advantage. In order to understand the paradoxical consequences of AI, this study builds a dual-path moderated mediation model based on the Job Demands-Resources (JD-R) paradigm.

**Methods:**

Using a two-wave longitudinal design with a 3-month interval and multi-source data from 250 experts in China, we combined survey measurements with quasi-experimental manipulations. The following findings were obtained using structural equation modeling (SEM) and bootstrapping.

**Results:**

(1) AI technology adoption is a job resource that increases Felt Obligation for Constructive Change (FOCC), but it also acts as a job demand that inhibits innovation by creating a sense of job insecurity; (2) task crafting is a crucial boundary condition that amplifies the positive mediation path while attenuating the negative pathway.

**Discussion:**

Based on the aforementioned findings, this study highlights the importance of considering employees' psychological states and behavioral changes while fostering technological innovation, exposing the intricacy of artificial intelligence technology in HRM from both a subjective and objective standpoint. Job insecurity is a possible drawback of technology use, hence businesses should take appropriate steps to lessen employee uneasiness while using new technologies. Felt Obligation for Constructive Change, on the other hand, is a crucial strategy for encouraging creative behavior. To do this, managers must investigate and enhance employees' intrinsic motivation for their everyday tasks and foster a culture of creativity. Task crafting, as an effective self-management and driving factor, is also very important to reduce the negative effects of technology adoption and increase its positive effects. For this reason, businesses should support and encourage employees to improve their autonomy and flexibility, iterate on their work methods, and stimulate their ability to innovate. This will not only help employees develop their own skills but also give businesses a competitive edge and continuous innovation motivation.

## 1 Introduction

At the beginning of the 21st century, enterprise digital transformation initiatives (Zhang and Wang, 2019) and strategic imperatives for optimizing operational efficiency have sparked the organizational proliferation of AI and service robotics (Liu and Xie, 2024). Human-AI symbiosis is a permanent organizational pattern, however, as ongoing technical limitations and operational complexity demand the continual use of human capital as key service agents (Hentout et al., 2019; Wang and Yao, 2022). Although AI implementation clearly improves procedural efficiency by automating repetitive tasks (Zhu et al., 2021), it also creates paradoxical workforce dynamics with human-AI integration conflicts (Wang and Yao, 2022). The techno-economic reorganization of labor allocation mechanisms has triggered occupational displacement anxieties (Liu and Xie, 2024), which show up as both productivity gains and career trajectory instability. This dualistic effect highlights the importance of explaining the psychobehavioral mechanisms behind AI's organizational penetration (Wang and Yao, 2022), especially its dualistic potential for work process augmentation and occupational identity disruption.

One important factor influencing business innovation performance is employee inventive behavior (Bao et al., 2024). This multifaceted construct represents a synergistic integration of psychological dispositions and behavioral manifestations (Fu et al., 2024). It includes both the proactive efforts to secure organizational support for the implementation of new, value-creating ideas or solutions as well as the generation of such ideas or solutions during work processes (Scott and Bruce, 1994). The introduction of artificial intelligence (AI) has caused a paradigm change in organizational ecosystems, with silicon-based intelligent agents (AI-powered digital entities) and carbon-based human capital (biological workforce) now serving as the two main pillars of productive assets (Peng, 2023). Employees' complicated cognitive assessments during human-AI interactions have been prompted by recent technical breakthroughs, especially with relation to the perception of AI as either an adversary or an ally that could promote collaboration (Liu and Xie, 2024).

Subjective psychological states and objective organizational circumstances interact dynamically to modulate employee innovative behavior. These interactions can either increase or decrease intrinsic motivational drivers, change innovation behavioral patterns, and ultimately have a significant impact on corporate innovation trajectories (Fu et al., 2024; Liu and Xie, 2024). There are still major gaps in understanding the contextualized mechanisms through which individual psychological constructs and organizational environmental factors differentially shape innovation processes across industrial sectors, despite the fact that existing research has made some progress in identifying generic antecedents of innovative behavior (Luo et al., 2022; Wang and Zhou, 2024). Additionally, the majority of current research focuses on how employees adjust to required human-AI collaboration requirements (Wang and Yao, 2022), ignoring systematic research into the endogenous psychological mechanisms and behavioral repertoires that result from proactive employee-initiated collaboration with AI systems (Wang and Zhou, 2024).

This study conducts a detailed analysis of the education and training sector using the dual-path framework of the JD-R model as its analytical lens in order to fill in these theoretical gaps. Our study aims to clarify the behavioral outcomes and processual processes that define employee adaptation to human-AI collaboration paradigms when multifactorial subjective predispositions and objective environmental circumstances are combined.

The widespread adoption of robotic systems and automated production technologies in organizational settings is a result of the development of digital technologies such as big data, cloud computing, and artificial intelligence (Li et al., 2021). AI has become an essential catalyst for organizational innovation as a result of this technical advancement. However, human capital continues to be the primary driver of business innovation, requiring careful research into how workplace AI integration influences creative behavior on an individual basis (Zhang et al., 2023). Positively, occupational features have been fundamentally reconfigured due to the emerging complexity of interpersonal coordination and human-machine collaboration (Yang and Qiu, 2020). Employees who are exposed to new technological paradigms are able to rethink the value of their work, find existential meaning in their work, and achieve psychological fulfillment through increased perceived competence—all of which are essential precursors that stimulate workplace engagement and encourage innovative approaches to problem-solving (Zhu et al., 2021). The chance of breakthrough innovations is significantly increased by this strategic resource release, which permits concentrated investment of intellectual and temporal capital into innovation-centric activities (Verma and Singh, 2022). On the other hand, the widespread use of robotics creates a sense of job insecurity, causing workers to think about their job security— a mentality that consistently deters risky, creative endeavors (Wang et al., 2019). Additionally, AI-driven efficiency requirements increase technostress through AI replacement anxiety and timing demands, which over time weakens perceived organizational support and stifles creative tendencies (Mirbabaie et al., 2022).

In order to optimize the human-AI symbiosis, it is crucial to intentionally increase AI's capacity to foster inventive consciousness and capabilities while reducing its psychological externalities. This dual-nature impact highlights this necessity. The key factor in maximizing AI's potential for innovation is striking a balance between technology enhancement and human-centered work design. According to Zhang et al. (2023), the JD-R model offers a useful framework for incorporating the contradictory effects of AI technology applications on creative employee behavior. According to this theoretical framework, workplace demands that exhaust psychological resources and job resources that promote motivational benefits are two different aspects of a job (Zhang et al., 2010). Employee task processes, methodologies, and content structures are naturally reconfigured by the integration of AI in the workplace—basic changes in job characteristic configurations (Man Tang et al., 2022; Mirbabaie et al., 2022). The dual-path process, in which work qualities exert opposing influences through gain and loss pathways, is a key component of the JD-R model (Demerouti et al., 2001). As demonstrated by psychological concepts like FOCC, job resources are constructive stimuli that promote resource accumulation and personal development (Bakker et al., 2023). In contrast, job demands are detrimental stimuli that lead to psychological exhaustion due to things like AI-induced job insecurity (Mirbabaie et al., 2022). Through work automation, AI deployment produces residual cognitive resources from a gain standpoint (Man Tang et al., 2022; Verma and Singh, 2022). Workers may purposefully devote their freed time to self-directed learning and the application of new skills, increasing professional responsibility and stimulating creative behavioral outputs. The loss viewpoint highlights the disruptive potential of AI: advances in technology threaten occupational status, professional identity, and skill obsolescence while imposing new competency requirements for workflows enhanced by AI (Wang et al., 2019). In the end, this combined pressure suppresses inventive behavior through increased job insecurity mechanisms (Huang and Li, 2016), which show themselves as technological displacement threats and creative destruction challenges (Wang et al., 2023). Importantly, individual views of job needs and resources show how work control dynamics are contingent (Karasek, 1979). One behavioral method to improve person-job congruence and occupational meaningfulness is task crafting, which is described as proactive changes to task scope, diversity, and execution modalities (Yin and Liu, 2016). High task-crafting propensity individuals show greater agency in rearranging job demand-resource equilibria, which may mitigate workplace issues brought on by AI (Bakker and Oerlemans, 2019). As a result, this study examines task crafting's moderating impacts on the dual-path outcomes of workplace AI integration, positioning it as a crucial boundary condition.

## 2 Literature review

### 2.1 Theoretical framework

#### 2.1.1 Job demands-resources model and AI

Job demands that require prolonged physical or psychological expenditure (e.g., employment precarity, temporal constraints) and job resources that reduce psychophysiological costs while promoting developmental trajectories (e.g., organizational support, professional accountability) are the two categories of occupational characteristics that the JD-R model, which is the theoretical foundation of this study, postulates (Demerouti et al., 2001). According to Karasek (1979), the JD-R framework mechanistically promotes a dual-process paradigm that emphasizes concurrent gain-loss dynamics in work situations. According to the motivational pathway, having a lot of job resources improves work engagement by building up psychological resources, which lessens burnout symptoms and produces positive behavioral results. On the other hand, the depletion route describes how high job expectations and insufficient resource availability lead to chronic stress, which in turn causes poor psychological health and unproductive work habits (Demerouti et al., 2001). The JD-R model has been empirically validated in a variety of industrial scenarios due to its structural versatility. Both of its core claims—the resource-driven "motivational process" with goal-directed energy mobilization and the demand-induced "health impairment process" with cumulative resource erosion— are supported by substantial research (Mudrak et al., 2018). Because of its theoretical flexibility, the model is especially well-suited to studying intricate organizational processes with contradictory results.

The term "workplace integration of AI technology" refers to the use of intelligent systems (such as robotic process automation, machine learning architectures, and speech recognition algorithms) that can learn on their own, reason logically, solve problems, and make decisions in order to improve the efficiency of task execution (Man Tang et al., 2022). The adoption of AI is conceptualized in this study as a catalyst for the metamorphosis of job characteristics (Craig et al., 2019), hence redefining the operational workflows and task architectures of employees (Wang et al., 2019). This dual-aspect shift implies that changes in job characteristics brought forth by AI have two separate effects on creative behavior via different JD-R pathways. AI releases cognitive surplus that strengthens employees' belief in organizational reform projects by mimicking human heuristic processes to accomplish procedural problems (Mirbabaie et al., 2022). By encouraging positive energy investment in systemic issue solving and procedural improvement, this resource accumulation amplifies creative behavioral consequences (López-Domínguez et al., 2013). On the other hand, the loss pathway shows up as a paradigmatic job demand. AI-induced employment precarity. As AI replaces traditional cognitive labor, workers face the possibility of technological redundancy, which increases job insecurity (Zhu et al., 2021; Wang et al., 2019).

Employee perceptions of job demands and resources are influenced by the extent of individual control over tasks (Karasek, 1979). Yin and Liu (2016) define task shaping as proactive adjustments to the amount, nature, and methods of work completion, such as adding new tasks to best utilize individual skills. Workers that have more authority over their work can better govern AI technology adoption and its results. This study conceptually bases task crafting as a crucial contingency factor mediating AI's paradoxical innovation impacts. First, people who have a high degree of task-shaping pay close attention to whether their current work status aligns with their personal traits when interacting and collaborating with computers (Downes et al., 2021). Consequently, in the context of applying AI technology, when workers with a high degree of task shaping discover a discrepancy between their abilities and the actual job roles, they will take subjective initiative, modify tasks on the fly, and adjust to the surroundings, which will increase job resources and lower job demands. Secondly, employee task structuring can greatly improve the work experience, help reinterpret the purpose of work, and elicit good feelings (Chen et al., 2014). High task shaping individuals have more control over their work, which enables workers to fulfill professional requirements and develop a sense of self-worth. As a result, they see AI technology as a job resource that facilitates rather than hinders job demands (Zhang et al., 2023).

#### 2.1.2 Felt obligation for constructive change

A multidimensional motivational framework that connects prosocial responsibility schemas with innovation agency is called "Felt Obligation for Constructive Change" (FOCC) (Zhu et al., 2023). FOCC operationalizes employees' self-regulated dedication to organizational improvement through extra-role initiative-taking and systemic problem-solving behaviors, as conceptualized by reciprocal determinism theory (Zhou and Qian, 2023b). The dual-layered duty that results from this cognitive-affective condition is (1) metacognitive awareness of stewardship imperatives and (2) behavioral intentionality toward actions that create value (Li et al., 2023). By internalizing systemic innovation as a moral need rather than a voluntary contribution, FOCC, which is theoretically based in psychological ownership frameworks (Eisenberger et al., 2001), goes beyond conventional organizational citizenship.

#### 2.1.3 Job insecurity

Shoss (2017) operationalizes job insecurity as a multifaceted construct: qualitatively as relational contract disintegration in human-capital ecosystems, and quantitatively as perceived disruption of employment continuity. Through algorithmic displacement processes, the emergence of AI-driven workplace change intensifies this techno-stress assessment, as workers cognitively rebuild intelligent automation as an existential danger to the preservation of occupational identity (Tu et al., 2023). Job insecurity, which is based on Bandura's agentic-cognitive appraisal processes, takes the form of three threat simulations: (1) a change in employment status, (2) a devaluation of professional capital, and (3) a breach of the psychosocial contract. Each of these scenarios sets off different neurocognitive stress pathways (Yang and Lu, 2023). According to this rethinking, the key psychosocial transmission mechanism behind AI-induced work precarity is job insecurity.

#### 2.1.4 Task crafting

Experts in job crafting Task, relational, and cognitive crafting are the three categories into which Wrzesniewski and Dutton divide job crafting (Yin and Liu, 2016). For the first time, job crafting is clearly described as a set of proactive actions taken by employees themselves with the goal of coordinating their passions, interests, and motivations with their work. By doing so, work cognition, relationship boundaries, and job tasks are altered (Hu and Tian, 2015). One of them, task creation, describes proactive adjustments to the amount, nature, and methods of work completion, like adding new activities to best utilize individual skills (Yin and Liu, 2016). The process of task crafting is goal-driven and entails both proactive goal-setting and proactive goal-achieving.

### 2.2 Research status

Organizations nowadays face a number of difficulties, including the rapid advancement of science and technology and the heightened competitiveness in the market. In order to preserve their competitive advantages, businesses are compelled to implement intelligent transformation. Mastering special and difficult-to-replicate resources and competencies is the key for firms looking to improve their core competitiveness. Organizational growth can be specifically supported by ongoing investment in and accumulation of AI innovation. As a result, AI technology has emerged as a vital and essential engine for organizational growth. Existing research has first validated the various benefits of AI innovation for firms from an organizational standpoint:

First, an organization's own capacity for innovation can be greatly enhanced by AI innovation (Xu et al., 2025). An organization can attain improved performance and more efficient operations when it incorporates AI innovation technology into its operations (Huang et al., 2022). A company's ability to absorb information, make decisions, and swiftly modify its plans in order to conform to the current industrial environment can all be improved by AI.

Secondly, AI innovation can lower operational expenses and resource waste while simultaneously increasing management efficiency through the optimization of production processes and management measures (Yan et al., 2025). AI-powered water-saving operation systems, for instance, can lower the amount of water and electricity used by businesses. Furthermore, cloud computing and autonomous learning-based AI technologies can assist firms in precisely identifying foreign investment risks and increasing the scope of opening-up (Zhang and Li, 2021).

Thirdly, AI innovation gives companies the chance to collaborate on new ideas and pool resources. For instance, it facilitates more effective cooperation and innovation in logistics firms (Huang et al., 2022). It should be highlighted, nevertheless, that the advantages that businesses derive from AI primarily rely on how well staff embrace and adjust to the technology. Employees' efficient use of the resources AI releases must be the foundation for AI's potential to increase an organization's capacity for creativity and operational efficiency. The beneficial value of AI for the company will be indirectly diminished if employees react negatively to it, creating a "AI-Employees-Organization" chain influence mechanism.

However, current research on AI's effects still has clear gaps and limits and has not yet developed an integrated viewpoint (Zhang et al., 2025). From a research coverage standpoint:

On the one hand, prior research in the field of organizational innovation has mostly concentrated on the influence of conventional elements like social networks, top management teams, organizational strategies, organizational structure and scale, and entrepreneurial diffusion (Damanpour, 2010; Baldridge and Burnham, 1973). Emerging AI technology has received less attention in the process of organizational innovation formation (Ru et al., 2025). Amabile (2020), a researcher in the field of creativity research, has explicitly asked for greater focus on the reciprocal relationship between AI, innovation, and creativity.

On the other hand, the issue of "perspective fragmentation" plagues current research, even when it comes to the effects of AI on people. Relevant research either focuses on the organizational level, examining how AI affects innovation resilience (Hou and Liu, 2024), innovation models (Chen et al., 2024), innovation performance (Wang, 2023), and transformation and upgrading in enterprises, while neglecting the individual innovation of micro-level employees, which is the primary driver of organizational innovation; or, despite focusing on the individual level, it primarily examines the single impact of AI according to a single logic: some studies highlight the positive empowerment of AI (Liang et al., 2025). By altering occupational skill requirements, for example, Zhu et al. (2021) suggested that AI improves workers' perception of vitality and competence. According to other research, AI can share repetitive tasks to increase workers' psychological availability (Kahn, 1990) and productivity (Verma and Singh, 2022), as well as decrease mechanical labor, freeing up employees' time to concentrate on creative work and increasing the likelihood of creative accomplishments (Verma and Singh, 2022). However, some studies also highlight the drawbacks of AI. For instance, Wang et al. (2019) examined how workers' job insecurity was affected by the widespread use of industrial robots. According to Yam (Yam et al., 2023), the introduction of robot labor would make workers feel insecure, which is a precursor to job burnout and barbaric actions. Additionally, studies show that the high demands of AI on productivity and workload will lead to increased time pressure, fear of replacement, a diminished sense of organizational support, a stifling of creative behavior, and even a "technological trap" and loneliness at work (Ozcelik and Barsade, 2018). The "double-edged sword" effect of AI technology on employees' innovative behaviors from two perspectives is not fully revealed by this research approach that focuses on just one effect (Ye et al., 2025). It also fails to acknowledge that both positive and negative employee-level reactions will be indirectly transferred to the organizational level, influencing the ultimate outcome of AI-driven organizational innovation and transformation.

The investigation of the influence mechanism of AI on current accomplishments is still insufficiently detailed from the standpoint of the breadth of research content. Relevant research largely ignores the crucial function of boundary circumstances in favor of concentrating on the direct and mediated effects of AI on businesses or workers (Wu et al., 2023). They do not go into great detail about what can make AI more beneficial to workers and, consequently, to the business, or how to mitigate its negative effects and prevent roadblocks to organizational growth. It is difficult to respond to the fundamental question of "how to balance the advantages and disadvantages of AI, and while exerting its organizational empowerment value, maintain employees' innovative awareness and capabilities" as a result of this incomplete examination of the "black box" mechanism of AI acting on the "employee-organization" chain. In conclusion, while AI has been shown to be a major force behind the intelligent transformation of organizations, and previous research has briefly discussed the dual effects of AI on employees and organizations, it has not thoroughly examined the function of AI from the integrated viewpoint of "organizational needs—employee reactions—organizational outcomes. The research viewpoint, thinking, and content depth can all be improved, and more thorough study is desperately needed to close the gaps.

## 3 Research hypotheses and model

### 3.1 AI technology adoption and felt obligation for constructive change

By taking on supra-role tasks, FOCC exemplifies employees' prosocial behavioral tendency to freely devote discretionary effort toward company progress (Yang et al., 2016). According to the JD-R paradigm, this construct is a quintessential job resource—a motivating agent that improves performance results and occupational efficacy. This study hypothesizes that the two methods of cognitive surplus liberation and resource accumulation are how workplace AI integration positively activates employees' FOCC. AI serves as a high-level source of job resources in this study because it can automate tasks and provide cognitive support, which lowers job demands and indirectly frees up employees' personal resources. According to the central tenet of the JD-R theory, which holds that resources that operate as a dynamic knowledge base stimulate positive psychological moods and behaviors, it also directly offers new instrumental resources, improving employees' perceptions of resource availability. Mechanistically, AI systems provide real-time visual insights while automating repetitive, automated, and cognitively demanding activities (Mikalef and Gupta, 2021). AI's automation capabilities reduce employees' cognitive load and mental exhaustion by taking over repetitive jobs that demand a lot of attentional resources. Employees feel both "resourceful" and "empowered" as a result of the mental resources released from such required duties, which create a "cognitive surplus" because individual cognitive capacity is restricted. Their proactive reinvestment of effort into methodological innovation and FOCC is contingent upon the perceived availability of cognitive resources. By reducing cognitive load demands, this technological replacement effect frees up temporal-spatial flexibility, allowing for methodological innovation and autonomous task reconfiguration (Man Tang et al., 2022). Perceptions of accountability are heightened by such operational autonomy, which increases perceived professional agency. At the same time, AI acts as a dynamic knowledge base that actively selects and contextualizes educational materials in line with workers' growth goals (Mikalef and Gupta, 2021).

Employees benefit from increased extra role self-efficacy through AI-enabled skill acquisition, which answers the perceptual query of "whether they can do it." The motivational question of "whether they are willing to do it" is addressed when they ascribe this improvement to organizational support in the form of AI resources, which evokes a strong sense of reciprocal obligation based on social exchange norms. When combined, these mechanisms complete the shift from resource accumulation to proactive change willingness by converting objective skill resources into a strong belief that one is both capable and accountable for promoting positive change within the business. With the help of this clever scaffolding, workers follow self-regulated learning paths, gaining new skills through needs-based time management (Parker and Grote, 2022). This iterative upskilling procedure reinforces organizational reciprocity norms and role-efficacy views while facilitating heuristic problem-solving (Zhou and Qian, 2023a). Employees' FOCC is raised in tandem by this dual-resource improvement, which is operationalized through workload efficiency and cognitive augmentation. Thus, we hypothesize:

**H1:** AI technology adoption is positively correlated with FOCC.

### 3.2 AI technology and job insecurity

Job insecurity is defined as the powerlessness people have in a situation where their job is in danger and their negative outlook on the long-term nature of future employment (Zhang and Long, 2013). The JD-R model states that job demands are detrimental elements that drain workers' energy and physical and mental well-being; as a result, job instability is classified as a typical job demand. According to this report, employees will experience job instability as a result of the use of AI technology. On the one hand, traditional office models might become outdated as a result of new technologies or technological changes within organizational structures. The objective trend of "obsolescence of traditional models" may indicate a deeper psychological threat rooted in fear rather than only indicating that employees need to update their skills. A sense of "skill depreciation" and concern over "weakened role relevance" arise when workers believe that the procedures, work techniques, and even fundamental talents they have mastered are becoming quickly standardized, automated, or rendered obsolete by AI tools. One of the main characteristics of job insecurity is the feeling of doubt about one's worth and job security. According to the JD-R model, it serves as a contextual stressor that may exhaust workers' psychological reserves and promote a defensive rather than a contributing mindset. On the other hand, extreme changes in the workplace and technological advancements can have a substantial impact on people's survival and development, making workers more likely to experience job insecurity (Wang et al., 2019; Ma et al., 2022). By surpassing expected tasks or responsibilities (Mirbabaie et al., 2022), breaking employees' psychological defenses, creating a sense of unemployment crisis, and increasing job insecurity, AI technology has the potential to replace employees' positions, skills, and professional knowledge. AI technology can also think and execute tasks independently (Man Tang et al., 2022). However, the quick changes and iterations of AI technology will change corporate work processes, methods, and characteristics; restructure the tasks of current job positions; and increase employee acceptance and transition levels to new professional techniques and knowledge, increasing the cost of job transition for employees (Craig et al., 2019). Employees are acutely aware of this pressure and see it as a latent threat signal: if they do not pick up new skills and adjust to new procedures quickly, they may not only perform worse but may also be seen as a "cost burden" because they aren't meeting the organization's expectations for return on investment. This exacerbates their worries about job instability, or the stability of their position. Employee job insecurity increases as a result of their perception of job replacement impacts and unemployment threats brought on by human-computer interaction. Thus, we hypothesize:

**H2:** AI technology adoption is positively correlated with job insecurity.

### 3.3 The mediating role of felt obligation for constructive change

FOCC is a vital job resource that stimulates people's positive work engagement states and is based on the JD-R framework (Yang et al., 2016). Under the gain pathway, this construct mechanistically appears as two interconnected mechanisms:

Firstly, FOCC fosters conscientious organizational stewardship, in which staff members use their discretionary resources to show an agentic commitment to organizational improvement (Yang H. et al., 2016; Zhou and Qian, 2023b). Innovative behavioral repertoires are sparked by this motivational tendency, which directs cognitive bandwidth and temporal investments toward ideation processes and innovation implementation cycles (Zhang et al., 2023). Secondly, employees with more constructive duty demonstrate a greater ability to utilize AI-generated resource slack in AI-augmented work environments that demand increased creativity and socio cognitive skills (Zhu et al., 2021). People are naturally inclined to use their excess resources to purchase more resources. Employees who have resource redundancy are more willing and able to take on the possible risks that come with innovation. They convert abstract "flexibility" into concrete FOCC behaviors by reinvesting surplus cognitive resources into considering and experimenting with current work practices. These people strategically organize implementation resources, spread proto-innovations through organizational networks, and proactively start cross-hierarchical collaborations (Sun et al., 2018). Standard operating procedures (SOPs) are continuously improved by this iterative process, and recurrent innovation stimuli are produced by emergent workflow optimizations. FOCC is a pro-organizational intrinsic motivator that gives workers a sense of purpose and validation—the "why" behind creativity. The "how" of innovation is addressed by the cognitive surplus and skill resources obtained through prior AI-enabled empowerment, which provide the required capacities and means. When the two are combined, employees internalize innovation as a component of their role identity rather than seeing it as an extra-role responsibility. They consequently more actively use redundant resources to experiment with methodology and solve problems on their own, converting positive intentionality into concrete, creative results. The combination of these processes—cognitive resource mobilization and collaborative knowledge synthesis—makes FOCC a crucial intermediary in converting AI-driven job resources into innovative results. Thus, we hypothesize:

**H3:** AI technology adoption positively affects employee innovative behavior through FOCC.

### 3.4 The mediating role of job insecurity

One major cause of stress in the workplace is job uncertainty. Continuous job demands can exhaust an individual's energy and job resources, which can have a variety of detrimental effects on employees' psychological states and behaviors, according to the loss route (Sjoberg, 2010). AI-induced uncertainty pushes workers into a state of constant cognitive evaluation and hypervigilance, which subtly and persistently depletes their finite psychological reserves. Accordingly, the use of AI technology causes employment uncertainty, which in turn serves as a psychological stressor that drains workers' personal resources and initiates the JD-R model's "loss path." Based on the JD-R model, job demands consistently exhaust personal energy or job resources, leading to a sequence of unfavorable consequences (Zhang et al., 2023). Innovative behavior by employees need a secure and encouraging work environment to develop, and the behavior itself brings risk and uncertainty, which is reflected in the amount, quality, and completion of tasks (Scott and Bruce, 1994; Sun et al., 2018). Employees' psychological self-defense mechanisms and need for stability are triggered when they believe that their job security and stability are always in danger. This might show itself as resistance to change at work, a greater dependence on traditional work practices or conformist methods, and a decrease in taking risks and taking creative action (Sjoberg, 2010; Liu et al., 2022). Existing research shows that organizational environment changes can easily cause employees to feel anxious and under pressure from technological unemployment. This can lead them to shy away from proactive innovative behaviors to lower the risk of making mistakes and avoid difficult and high-risk tasks, which in turn can stifle their enthusiasm for innovation (Ma et al., 2022). A psychological process of risk reassessment is triggered by job instability. When workers perceive that their jobs are in danger, they stop considering the possible advantages of innovation and instead focus on the possible consequences of failure, such rapid obsolescence or harm to their reputation. They subjectively reclassify innovative behaviors as dangers as a result of their increased sensitivity to the cost of failure, which causes a behavioral shift toward risk aversion and self-defense tactics. Thus, we hypothesize:

**H4:** AI technology adoption negatively affects employee innovative behavior through job insecurity.

### 3.5 The moderating role of task crafting

The JD-R model's dual-path mechanism shows contingency upon people's locus of work control; occupational autonomy gradients inherently influence the intensity of perceived job demands and resources (Karasek, 1979). Given that task creating is the most immediate and logical primary coping method when employees face unfavorable job circumstances, this study especially looks at it as the focal moderator (as opposed to relational or cognitive crafting versions) (Lin et al., 2017). As a strategic response to changing job demands, task crafting is defined as the proactive reconfiguration of task scope, procedural sequences, and execution modalities to maximize skill deployment efficacy and recalibrate work engagement orientations (Yin and Liu, 2016; Parker and Grote, 2022). The advantages of AI as a work resource are maximized at a high level of task building, when employees proactively optimize task boundaries, methodologies, and relationships. It makes it possible for workers to more consciously direct the cognitive surplus that AI frees up into areas that they find personally fulfilling and supportive of their agency. (Dvorak, 2014) This immediately satisfies fundamental psychological requirements—like autonomy and competence—that propel positive feelings inside the JD-R model's gain pathway by amplifying the perception of resource acquisition and improving their sense of control and purpose at work. This study hypothesizes that task crafting uses two synergistic strategies to increase the gain pathway connecting AI integration to FOCC. Firstly, role identity salience—a cognitive schema that prioritizes proactive process optimization and responsibility assumption—is present in high-task-crafting employees (Du et al., 2022). Through increased internalization of accountability, this agentic orientation exacerbates FOCC (Yang et al., 2016; Yan and Hao, 2020). Secondly, these individuals purposefully practice developing competencies by utilizing AI-generated temporal slack and remaining cognitive resources (Ma and Zhang, 2024). Constructive duty is amplified by this competency-accretion cycle, which supports views of technological enablement—the idea that AI increases discretionary control over innovation trajectories (Zhang et al., 2023). Employees with poor task-crafting skills, on the other hand, exhibit role passivity, which is an excessive dependence on external support systems combined with work technique conservatism. Low task crafting levels force workers to follow a passive pattern of routine execution, giving AI systems more control over change. The development of FOCC is ultimately hampered by this division of responsibilities and resource underutilization, which splits the road from technological empowerment to proactive contribution. This operational inertia causes the spread of responsibilities, which gradually weakens the FOCC. Thus, we hypothesize:

**H5:** Task crafting positively moderates the relationship between AI technology adoption and FOCC.

Combining H1 and H3, a further moderated mediation hypothesis is proposed:

**H6:** The indirect impact of AI technology adoption on employee innovative behavior is mitigated by task crafting via FOCC.

According to this study, the loss pathway of AI technology adoption to job insecurity will be weakened by task crafting. First of all, when workers feel in charge of their work, job instability can be decreased, which encourages creative behavior (Zhu et al., 2021). Task crafting turns workers from passive consumers of technology into active designers of their work by giving them the freedom to proactively modify job boundaries and procedures. The primary source of perceived control is this sense of direct intervention in work processes, which lays the psychological groundwork for later reducing emotions of insecurity. Strong task crafting skills enable workers to collaborate with AI technology and function as "captains" of their work, keeping them competitive with intelligent robots and lowering job insecurity and replacement anxiety (Wang et al., 2019). Secondly, people with a high degree of task crafting actively modify and adapt current tasks, completing established work in a flexible and independent manner rather than strictly following outdated guidelines. This enables workers to discover work methods that work better for them and more successfully adjust to changing demands and environments (Wang and Yao, 2022; Ma et al., 2022). On the other hand, people with low task crafting skills rely too much on pre-existing tasks because they think that fulfilling fundamental work requirements and avoiding errors and risks are more important. This leads to a mismatch between their needs and the challenges and learning that come with using AI technology (Zhang et al., 2023). Employee work practices and the organization's AI-optimized requirements are significantly out of sync when task crafting is low. Employees who experience this adaptation lag become painfully aware of the widening gap between their own abilities and job needs, which directly translates "rigid work patterns" into uncertainty about their ongoing usefulness and, eventually, creates the feeling of unemployment risk: "Will technology take my place?" Employees perceive a higher danger of technical unemployment as a result of this sluggish work style, which heightens job insecurity. Thus, we hypothesize:

**H7**: Task crafting negatively moderates the relationship between AI technology adoption and job insecurity.

Combining H2 and H4, a further moderated mediation hypothesis is proposed:

**H8**: The indirect impact of AI technology adoption on employee innovative behavior is mitigated by task crafting through job instability.

**Figure 1:**
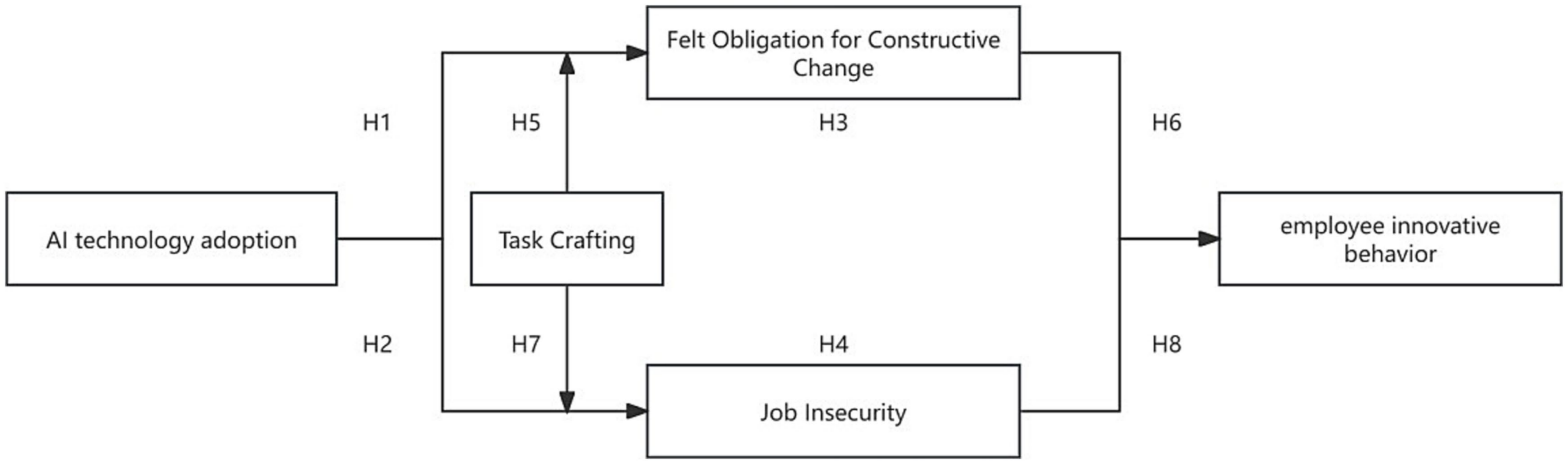
Theoretical model.

In conclusion, this study introduces the theoretical model depicted in Figure 1.

## 4 Study 1 method

### 4.1 Participants

Purposive sampling was used in Study 1 to choose staff members of Company C. The research subjects for this survey were formal workers who have been with the company for more than 6 months and whose daily work routines have been incorporated into the management of the AI system.

This company has demonstrated remarkable industry foresight by seamlessly integrating artificial intelligence technologies throughout all operational chains, including product development, user services, and organizational management. It is a leading player in Southwest China's online education sector and a representative example among emerging internet companies. Artificial intelligence adoption and impact mechanisms in professional environments can be studied in a highly condensed and representative real-world setting thanks to the company's vast coverage and deep use of AI. A dual-channel system (online platform + onsite administration) with procedural controls was used for data collecting. Prior to implementation, senior management formally approved the research through the involvement of institutional gatekeepers. The company's human resources department first sent out an online questionnaire link through the internal email list. A week later, the research team visited the company's headquarters and, with the help of department supervisors, arranged for employees who had not yet replied to the questionnaire to do so in a centralized on-site session. Every step of the data collection process was closely monitored in order to finish it in 2 weeks. Strict confidentiality rules protecting corporate data sovereignty were made clear to participants about the study's two goals: (a) mapping adoption trends of AI technology in workplace operations and (b) investigating psycho-behavioral adaptations after AI implementation.

In accordance with accepted methodological antecedents, the survey questionnaire included operational definitions of workplace AI technology adoption to guarantee ecological validity. To improve response fidelity, participants were given digital red envelopes with randomized cash incentives (CNY 1-2) after finishing the survey. Questionnaires having a response time of less than 30 s, duplicate responses, and illogical reasoning were eliminated after data filtering. With an efficient recovery rate of 89.93%, 250 valid questionnaires out of the 278 that were initially issued were ultimately kept. The final sample included 42% male and 58% female participants (M-age = 27.96 years), with 46.4% of participants being between the ages of 18 and 25, as shown in Table 1. 33.2% of employees had 1–5 years of organizational experience, according to a workforce tenure analysis (M-tenure = 3.88 years). Table 1 give complete demographic distributions in a systematic manner.

**Table 1:** Overall frequency analysis.

Name	Option	Frequency	Percentage (%)	Cumulative percentage (%)
1. Gender	Male	105	42	42
	Female	145	58	100
2. Age	Under 18	18	7.2	7.2
	18–25 years old	116	46.4	53.6
	26–30 years old	53	21.2	74.8
	31–40 years old	38	15.2	90
	41–50 years old	12	4.8	94.8
	Over 50	13	5.2	100
3. Education Level	High school or below	12	4.8	4.8
	Associate degree	23	9.2	14
	Bachelor's degree	97	38.8	52.8
	Master's degree	74	29.6	82.4
	Doctoral degree or above	44	17.6	100
4. Years of Employment	Internship period	43	17.2	17.2
	Probation period	22	8.8	26
	Within 1 year	36	14.4	40.4
	1–3 years (excl. 3 years)	59	23.6	64
	3–5 years (excl. 5 years)	24	9.6	73.6
	5–10 years (excl. 10 years)	41	16.4	90
	Over 10 years	25	10	100
	Total	250	100.000	100.000

### 4.2 Measurement

Western measurement tools with proven validity and reliability were used in this study. 5-point Likert scales with 1 denoting "strongly disagree" and 5 denoting "strongly agree" were used to measure each construct. In particular, Man Tang et al.'s (Man Tang et al., 2022) scale evaluating employee-AI interaction dynamics was used to evaluate AI technology adoption. A sample statement reads "My organization has implemented substantial AI technologies and equipment that influence multiple work dimensions, including reasoning, decision-making, and problem-solving processes" (α = 0.87). FOCC was operationalized through Eisenberger et al.'s (Eisenberger et al., 2001) scale, reflecting employees' felt duty to assist organizational improvement through positive changes. Representative items include "I consider it my obligation to contribute to organizational change and development" (α = 0.94). The Mauno et al.'s (2001) item "I worry about being compelled to resign before voluntarily leaving my position" (α = 0.73) was used to measure job insecurity. The task crafting sub dimension of Bindl et al.’s (2019) job crafting scale served as the basis for the task crafting evaluation. "I regularly incorporate preferred elements into my work responsibilities" (α = 0.90) is a characteristic item. The Zhang et al.'s (2016) measure measuring willingness to apply creative job adjustments was used to assess innovative behavior among employees. "I frequently experiment with novel approaches to resolve workplace challenges" (α = 0.844) is one example item.

## 5 Study 1 results

### 5.1 Preliminary data analysis

According to Table 2, the four dimensions of AI technology adoption, FOCC, job insecurity, and employee inventive behavior all match psychometric norms for internal consistency (Cronbach's α > 0.80) and composite reliability (CR > 0.80). Meanwhile, this study used AMOS 24.0 software to test the overall fitness of variables. According to the calculation results of fitness parameters shown in Table 3: x^2^/df = 1.444 < 3, GFI = 0.906 > 0.9, AGFI = 0.895 > 0.8, CFI = 0.9720.9, RMSEA = 0.033 < 0.05, SRMR = 0.037 < 0.08, NFI = 0.906 > 0.9. All indicators met the fitness criteria, indicating that the discriminant validity was established. Convergent validity is also confirmed by AVE values that exceed the 0.50 threshold for all constructs. Significant item intercorrelations (KMO = 0.88, *p* < 0.05) in the factor analysis show methodological rigor and validate the structural validity of the measurement model.

**Table 2:** Results of questionnaire reliability and validity analysis.

Item		Item-deleted α coefficient	Cronbach's α	AVE	CR
AI technology adoption	A1	0.876	0.836	0.573	0.841
	A2	0.878			
	A3	0.878			
	A4	0.880			
FOCC	B1	0.863	0.866	0.612	0.829
	B2	0.863			
	B3	0.865			
	B4	0.864			
	B5	0.862			
Job insecurity	C1	0.863	0.819	0.623	0.882
	C2	0.867			
	C3	0.864			
Task crafting	D1	0.864	0.842	0.582	0.846
	D2	0.863			
	D3	0.865			
	D4	0.864			
Employee innovative behavior	E1	0.866	0.865	0.529	0.869
	E2	0.864			
	E3	0.864			
	E4	0.863			
	E5	0.867			
	E6	0.862			

**Table 3:** Correlation analysis results for various dimensions.

AI		FOCC	Job insecurity	Task crafting	Employee innovative behavior
AI technology adoption	1***				
FOCC	0.358***	1***			
Job Insecurity	0.394***	−0.717*** 0.384***	1***		
Task Crafting	0.328***		−0.377***	1***	
Employee Innovative Behavior	0.278***	0.404***	−0.361***	0.852***	1***

*p < 0.05, **p < 0.01, *** *p* < 0.001, *N* = 250.

This study uses SPSS 27.0 to do correlation analysis, and the correlation analysis findings are shown in Table 4. It is evident that AI has a strong positive correlation with both FOCC (r = 0.358, *p* < 0.001) and job insecurity (r = 0.394, *p* < 0.001). Furthermore, in line with the theoretical hypotheses, there is a significant negative correlation (r = −0.361, *p* < 0.001) between job insecurity and employee innovative behavior, and a significant positive correlation (r = 0.404, *p* < 0.001) between FOCC and innovative behavior.

**Table 4:** Recommended values and actual values of model fitness.

Fitness Index	x^2^/df	GFI	AGFI	CFI	RMSEA	SRMR	NFI
Recommended Value	<3	>0.9	>0.8	>0.9	<0.05	<0.08	>0.9
Actual Value	1.444	0.906	0.895	0.972	0.033	0.037	0.906

### 5.2 Hypothesis testing

The results of the hypothesis testing were methodically recorded in Tables 5 and 6, and SPSS 27.0 was used for statistical analysis in this study. H1 is confirmed by the analytical results, which show a substantial positive connection between AI technology adoption and FOCC (β = 0.351, *p* < 0.001). At the same time, there is a significant positive link between the use of AI technology and job insecurity (β = 0.392, *p* < 0.001), which empirically supports H2. Notably, employment instability exhibits a substantial negative correlation with inventive activity (β = −0.367, *p* < 0.001), but FOCC positively predicts such conduct (β = 0.421, *p* < 0.001). Key status factors (gender, age, and occupational tenure) were analytically divided using covariance stratification, adhering to quasi-experimental design norms, in order to separate the AI-perception variance that may be attributed to techno-psychological mechanisms from false demographic covariance.

**Table 5:** Regression analysis of AI technology adoption on FOCC and job insecurity.

Variable	FOCC				Job Insecurity			
	Model 1		Model 2		Model 3		Model 4	
	B	T	B	T	B	T	B	T
Gender	0.100	1.589	0.106	1.793	0.041	0.645	0.048	0.810
Age	0.024	0.360	0.003	0.046	0.067	0.990	0.043	0.693
Education Level	0.110	1.629	0.057	0.900	0.087	1.277	0.029	0.448
Years of Professional Experience	−0. 142	−2.183	−0.118	−1.919	−0.003	−0.039	0.025	0.411
AI technology adoption			0.351	5.885 ***			0.392	6.609 ***
F-value	2.065		8.805		0.599		9.298	
R-squared	0.033		0.153		0.010		0.160	

*p < 0.05, **p < 0.01, ***p < 0.001, *N* = 250.

**Table 6:** Regression analysis of FOCC and job insecurity on employee innovative behavior.

Variable	Employee innovative behavior							
	Model 5		Model 6		Model 7		Model 8	
	B	T	B	T	B	T	B	T
Gender	−0.040	−0.630	−0.083	−1.407	−0.040	−0.630	−0.056	−0.928
Age	0.009	0.127	−0.002	−0.025	0.009	0.127	−0.016	−0.252
Education Level	−0.027	−0.397	−0.073	−1.171	−0.027	−0.397	−0.059	−0.932
Years of Professional Experience	−0.017	−0.253	0.043	0.709	−0.017	−0.253	−0.016	−0.256
FOCC			0.421	7.126 ***				
Job Insecurity							−0.367	−6. 142 ***
*F*-value	0.174		10.323		0.174		7.706	
*R*-squared	0.003		0.175		0.003		0.136	

*p < 0.05, **p < 0.01, ***p < 0.001, *N* = 250.

The effect size of "AI Technology adoption → FOCC → Employee Innovative Behavior" in the job resources route is 0.106, with a 95% CI of [0.057, 0. 161], as indicated in Table 7. This suggests that AI technology has a large favorable impact on employee inventive behavior through FOCC, supporting H3. The effect size of "AI Technology Application → Job Insecurity → Employee Innovative Behavior" in the job demands route is −0.103, with a 95% confidence interval of [−0.157, −0.049]. This suggests that AI technology has a considerable detrimental impact on employee inventive behavior through job insecurity, supporting H4.

**Table 7:** Model regression coefficients

Path			β	SE	t	P
AI technology adoption	→	FOCC	0.351	0.058	5.885	0.000***
AI technology adoption	→	Job Insecurity	0.392	0.065	6.609	0.000***
FOCC	→	Employee Innovative Behavior	0.421	0.058	7.126	0.001***
Job Insecurity	→	Employee Innovative Behavior	−0.367	0.051	−6.142	0.000***

**Figure 2:**
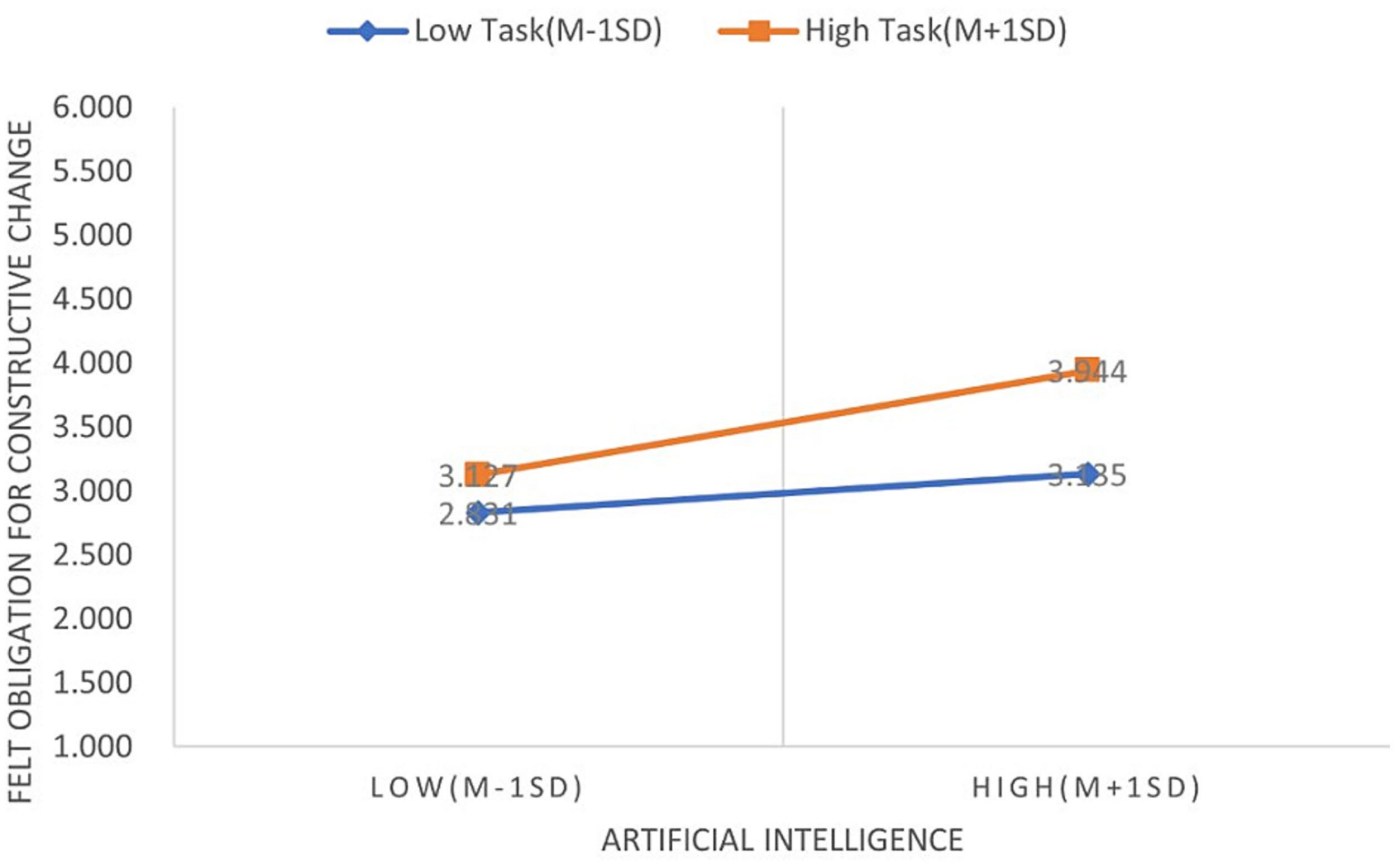
The moderating role of task crafting in the relationship between AI technology adoption and FOCC.

**Figure 3:**
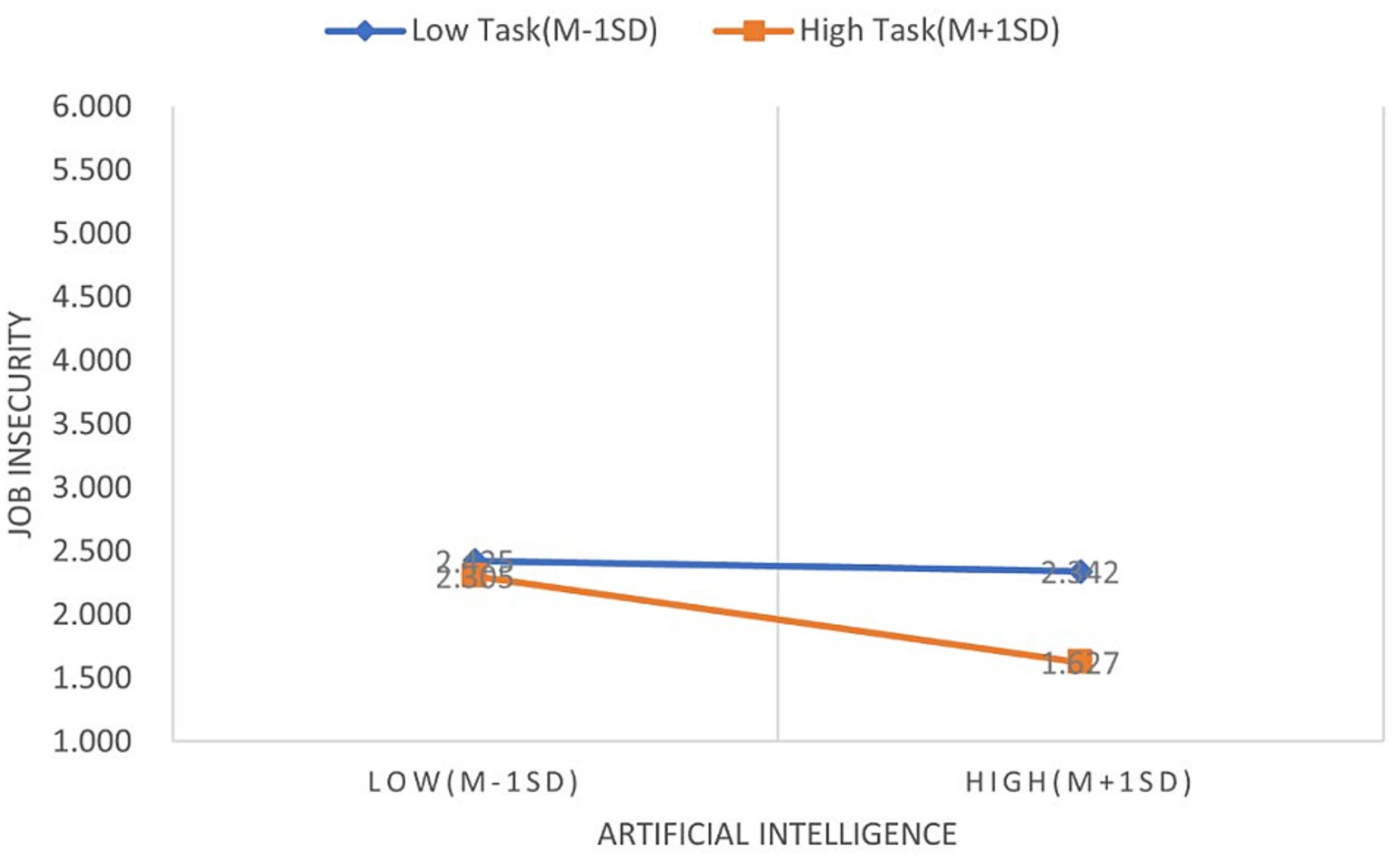
The moderating role of task crafting in the relationship between AI technology adoption and job insecurity.

H5 is confirmed by the interaction effect between task crafting and AI technology adoption, which shows a substantial positive influence on FOCC (β = 0.102, *p* < 0.1), as shown in Table 8. H7 is empirically supported by this interaction term, which shows a statistically significant negative correlation with job insecurity (β = −0.192, *p* < 0.001). Simple slope analyses were performed to visually represent the moderating function of task creation, and the findings are shown in Figures 2, 3. In particular, Figure 2 shows that the positive correlation between the use of AI technology and FOCC is stronger when task crafting is high as opposed to low. Similarly, when task crafting is increased, Figure 3 shows a larger negative correlation between AI adoption and job insecurity.

**Table 8:** Correlation coefficients for mediation effect analysis.

Effect	Path relationship	Effect Size	Standard error	Lower	Upper bound
Indirect Effect	Lnd: X → M1 → Y	0.106	0.026	0.057	0.161
	Lnd: X → M2 → Y	−0.103	0.027	−0.157	−0.049
Direct Effect	X → Y	0.063	0.058	−0.051	0.177
Total Effect		0.066	0.062	−0.057	0.289

AI technology adoption = X; FOCC = M1; Job Insecurity = M2; Employee Innovative Behavior = Y.

**Figure 4:**
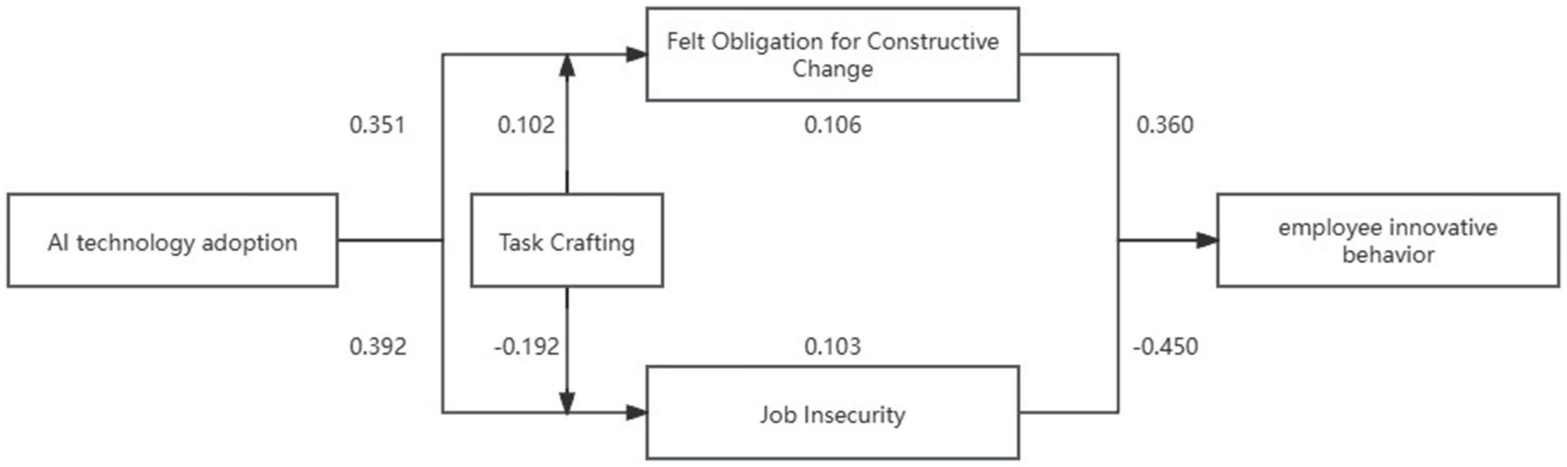
Path coefficient diagram of the research model.

The moderated mediation analysis of Model 7 showed clearly diverse patterns among the mediating factors, as indicated in Table 9. The indirect effect through FOCC was 0.138 (Boot SE = 0.085) at low levels of task crafting and was not statistically significant (95% Bootstrap CI = [−0.028, 0.305]); at high levels of task crafting, the indirect effect increased to 0.360 (Boot SE = 0.081) and became statistically significant (95% Bootstrap CI = [0.201, 0.519]). This moderating effect of task crafting supported Hypothesis H6 when family-organizational cultural congruence (FOCC) was included as a mediator. A similar moderating pattern was seen when job insecurity was tested as a parallel mediator: at low task crafting levels, the indirect effect through job insecurity was −0.09 (Boot SE = 0.108) and remained non-significant (95% Bootstrap CI = [−0.304, 0.123]); however, as task crafting increased, the indirect effect via job insecurity strengthened to −0.45 (Boot SE = 0.103) and became statistically significant (95% Bootstrap CI = [−0.662, −0.255]), empirically supporting Hypothesis H8. When combined, these disparate patterns emphasize how crucial it is to set boundary requirements in dual-mediation frameworks. Additionally, as seen in Figure 4, a path coefficient diagram was created to graphically display parameter estimates for every pathway.

**Table 9:** Moderated regression results for the moderating effect of task crafting.

Variable	FOCC						Job insecurity					
	Model 9		Model 10		Model 11		Model 12		Model 13		Model 14	
	Β	T	Β	T	Β	T	Β	T	Β	T	Β	T
Gender	0.100	1.589	0.115	2.039	0.105	1.873	−0.041	−0.645	−0.055	−0.978	−0.038	−0.678
Age	0.024	0.360	0.002	0.041	−0.019	−0.308	−0.067	−0.990	0.043	−0.715	−0.003	−0.056
Education	0.110	1.629	0.089	1.468	0.081	1.331	−0.087	−1.277	−0.057	−0.934	−0.041	−0.686
Years of	−0. 142	−2.183	−0.135	−2.314	−0.131	−2.261	0.003	0.039	−0.010	−0.164	0.016	−0.286
AI technology adoption			0.243	4.046 _***_	0.239	3.998 _***_			−0.296	−4.887 _***_	−0.288	−4.869 _***_
Task Crafting			0.315	5.280 ***	0.309	5.194 ***			−0.282	−4.711 ***	−0.271	−4.612 ***
Interaction Term					0.102	1.785 *					−0.192	−3.393 ***
*F*-value	2.065		12.791		11.518		0.599		12.119		12.482	
*R*-squared	0.033		0.240		0.250		0.010		0.230		0.265	

*p < 0.05,**p < 0.01, ***p < 0.001, *N* = 250.

## 6 Study 2 method

### 6.1 Participants

Participants in Study 2 were recruited from a variety of functional sequences within Company C in the online education and training sector, including Administration (Fixed Assets), Administration (Employee Benefits), HR Recruitment, HR Employer Operations, HRBP, and Administration (Daily Operations) among full-time employees. The company's internal internet portal was used to distribute all of the scales used in this study at two different times, separated by 1 month. In particular, the second measurement (T2) was utilized to monitor the dynamic changes of pertinent core factors, whereas the first test (T1) primarily gathered demographic information and basic data. With a youth-skewed age stratification (74.44% ≤ 30 years) and nearly equal gender representation (47.78% male; 52.22% female), the post-screening analytical cohort (N = 90) reflected emerging worker demographics in technology-intensive industries.

### 6.2 Study design

For a one-month manipulation experiment, Study 2 used a controlled variable technique. Before assigning experimental conditions, pre-intervention baseline data collection was procedurally sequenced by administering a socio-technographic survey to create status characteristic profiles in accordance with experimental procedure standardization. In accordance with the methods of academics like Tang, the definition of AI technology adoption was given to the participants in order to help them differentiate between AI technology and conventional technologies used in the workplace. A set of initial dimension questionnaires was then given to the participants, which included five dimensions: AI technology, task crafting, job insecurity, FOCC, and employee inventive behavior. The experiment included 90 valid questionnaires were collected after eliminating ineligible samples. The purpose of the baseline data collection was to provide a comparable benchmark for further data analysis before the measured variables were altered. Lastly, the 90 individuals were divided into three experimental groups at random, each consisting of 30 people: the control group, experimental group 1, and experimental group 2. Random assignment guaranteed the validity of the study's findings and assisted in removing selection bias among personnel.

### 6.3 Measure

Study 2 employs the same questionnaire scales as Study 1. Participants in the control group (no use of AI vs. low task crafting) will offer an unbiased point of reference throughout the experiment, highlighting the fact that employees' own experience and knowledge base are more important for problem-solving than AI-assisted decision-making or problem-solving tools. Participants in experimental group 1 (use AI vs. low task crafting) are allowed to employ AI technology in their daily job, but they are not allowed to change the structure of task content or work procedures. Participants in experimental group 2 (use AI vs. high task crafting) are backed by AI technology and are free to actively modify the amount, procedures, and techniques of work to meet job needs. Following the experiment, discrepancies between the groups are found and interpreted using statistical techniques.

## 7 Study 2 results

### 7.1 Paired samples T-test

Tables 10–12 show the results of the manipulation checks that were performed in this study using paired samples T-tests. Prior to and following the experiment, there were no significant differences in the control group, as indicated by the p-values for the following dimensions: AI technology adoption (*p* = 0.173), job insecurity (*p* = 0.646), FOCC (*p* = 0.423), task crafting (*p* = 0.512), and employee innovative behavior (*p* = 0.409). The effectiveness of the intervention "varying degrees of task crafting" was demonstrated by the p-values for AI technology application (*p* = 0.035), job insecurity(*p* = 0.000), and employee innovative behavior (*p* = 0.027) in experimental group 1, as well as the p-value for FOCC (*p* = 0.054), which was less than 0.1 and indicated differences before and after the experiment; the p-value for task crafting (*p* = 0.141) was greater than 0.1, indicating no significant change in this dimension; and the p-values for AI technology adoption (*p* = 0.000), job insecurity (*p* = 0.043), and FOCC (*p* = 0.012) were all less than 0.05, as well as the p-value for employee innovative behavior (*p* = 0.062), which was less than 0.1 in experimental group 2.

**Table 10:** Analysis of moderated mediation effects.

Mediator variable	Conditional indirect effect					
	Level	Level value	Effect	Boot SE	Boot LLCI	Boot ULCI
FOCC	Low Level (−1SD)	2.558	0.138	0.085	−0.028	0.305
	Mean	3.290	0.249	0.582	0.134	0.364
	High Level (+1SD)	4.500	0.360	0.081	0.201	0.519
Job Insecurity	Low Level (−1SD)	2.558	−0.09	0.108	−0.304	0.123
	Mean	3.529	−0.27	0.075	−0.421	−0.128
	High Level (+1SD)	4.500	−0.45	0.103	−0.662	−0.255

**Table 11:** Paired sample T-test results for the control group before and after the experiment.

Paired variable	Mean ± standard deviation			t	*p*	Cohen's d
	Pair 1	Pair 2	Paired			
AI	2.6 ± 1.545	3. 1 ± 0.885	−0.5 ± 0.66	−1.397	0.173	0.255
Job Insecurity	3. 167 ± 1.177	3.333 ± 1.348	−0. 167 ± −0.171	−0.464	0.646	0.085
FOCC	3.467 ± 1.502	3.2 ± 1.126	0.267 ± 0.376	0.812	0.423	0.148
Task Crafting Employee	3.467 ± 0.973	3.267 ± 1.285	0.2 ± −0.312	0.665	0.512	0. 121
Innovative Behavior	3.433 ± 0.898	3.2 ± 1.243	0.233 ± −0.345	0.839	0.409	0.153

*p < 0.05, ** *p* < 0.01, *** *p* < 0.001, *N* = 90.

**Table 12:** Paired sample T-test results for experimental group 1 before and after the experiment.

Paired variable	Mean ± standard deviation			t	*p*	Cohen's d
	Pair 1	Pair 2	Paired			
AI	3.733 ± 1. 112	3. 167 ± 1.177	0.567 ± −0.065	2.207	0.035	0.403
Job Insecurity	3.767 ± 1.278	2.267 ± 0.907	1.5 ± 0.371	5.736	0.000	1.047
FOCC	3.467 ± 0.9	3. 1 ± 1.029	0.367 ± −0.129	2.009	0.054	0.367
Task Crafting	3.567 ± 1.04	3. 133 ± 1.224	0.433 ± −0.184	1.513	0. 141	0.276
Employee innovative behavior	3. 1 ± 0.885	3.667 ± 0.994	−0.567 ± −0.109	−2.332	0.027**	0.426

*p < 0.05, **p < 0.01, ***p < 0.001, *N* = 90.

### 7.2 Independent samples T-test

In order to evaluate baseline equivalency between experimental cohorts before experimental modification, this study used an independent-samples t-test; pre-post comparisons are methodically FOCC, task crafting, and employee innovative behavior—show nonsignificant between-group differences (*p* > 0.05), according to the statistical results. In particular, at the pretest stage, randomized samples from Experimental Cohort 1 vs. Cohort 2 and the control group vs. Experimental Cohort 1 showed similar demographic features. Prior to treatment delivery, these psychometric equivalencies guarantee uniformity across experimental conditions and validate the effectiveness of the randomization procedure.

According to post-experimental analyses, the control group and Experimental Group 1 showed statistically significant intergroup differences in four important aspects, as presented in Tables 13 and 14: employee inventive behavior (*p* = 0.020), job insecurity (*p* = 0.001), AI technology adoption (*p* = 0.001), and FOCC (*p* = 0.022). On the other hand, there was no discernible difference in the task crafting (*p* = 0. 116). This pattern indicates that when task crafting was consistently kept at low levels, the experimental modification of AI technology adoption successfully generated quantifiable behavioral and perceptual alterations between these groups. Additional comparison between Experimental Groups 1 and 2 revealed a trend-level difference in employee inventive behavior (*p* = 0.086), marginally significant variations in FOCC (*p* = 0.055), and significant differential impacts in job insecurity (*p* = 0.026). Even though all groups received identical AI technology adoption, these graded results show consistent differences that can be attributed to the differential task crafting intensity modification. The empirical convergence of these results supports the operational validity of Study 2's dual experimental manipulations (Table 15).

**Table 13:** Paired sample T-test results for experimental group 2 before and after the experiment.

Paired variable	Mean ± standard deviation			t	*p*	Cohen's d
	Pair 1	Pair 2	Paired difference			
AI	3.367 ± 1.129	2.267 ± 0.907	1. 1 ± 0.222	4.164	0.000***	0.76
Job	2.267 ± 0.907	2.533 ± 1.074	−0.267 ± −0.167	−2. 112	0.043**	0.386
FOCC	3.2 ± 1.031	3.967 ± 1.066	−0.767 ± −0.036	−2.677	0.012**	0.489
Task Employee	3. 1 ± 0.759	3.533 ± 0.86	−0.433 ± −0.102	−1.987	0.056*	0.363
Innovative behavior	2.967 ± 1.033	3.467 ± 1.137	−0.5 ± −0.103	−1.945	0.062*	0.355

*p < 0.05, ** *p* < 0.01, ***p < 0.001, *N* = 90.

**Table 14:** T-test results: control vs. experimental group (pre/post).

Variable name	Variable	Before			After		
		Standard deviation	T-Test	Mean difference	Standard deviation	T-Test	Mean difference
AI technology	Non-Use	1.627	T = −1.027	0.367	0.681	T = −3.507	0.783
	Use	1.085			1.017		
Job Insecurity	Non-Use	1.093	T = −1.342 *p* = 0.185	0.367	0.671	T = −3.545 *p* = 0.001 ***	0.745
	Use	1.022			0.935		
FOCC	Non-Use	1.251	T = 0.897 *p* = 0.373	0.267	0.552	T = −2.36 *p* = 0.022 **	0.42
	Use	1.042			0.803		
Task Crafting	Non-Use	0.973	T = 1.627 *p* = 0.109	0.367	0.792	T = −1.594 *p* = 0.116	0.317
	Use	0.759			0.747		
Employee Innovative Behavior	Non-Use	1.348	T = 0.793 *p* = 0.432	0.233	0.557	T = −2.409 *p* = 0.020 **	0.467
	Use	0.885			0.903		

*p < 0.05,**p < 0.01, ***p < 0.001, *N* = 90.

**Table 15:** T-test results: experimental groups 1 & 2 (pre/post).

Variable name	Variable	Before			After		
		Standard deviation	T-Test	Mean difference	Standard deviation	T-Test	Mean difference
AI technology adoption	Low Degree	1.017	T = 0.482 *p* = 0.631	0.133			
	High Degree	1. 122					
Job Insecurity	Low Degree	0.94	T = 0.244 *p* = 0.808	0.056	0.935	T = 2.288 *p* = 0.026	0.567
	High Degree	0.821			0.983		
FOCC	Low Degree	1.104	T = −1.204 *p* = 0.234	0.334	0.869	T = −1.959 *p* = 0.055	0.44
	High Degree	1.04			0.871		
Task crafting	Low Degree	1.006	T = −0.74 *p* = 0.463	0.167			
	High Degree	0.714					
Employee Innovative	Low Degree	0.757	T = −0 0.319 *p* = 0.751	0.061	0.907	T = −1.751	0.367
	High Degree	0.726			0.702	*p* = 0.086	

*p < 0.05,**p < 0.01, ***p < 0.001, *N* = 90.

While outlining the crucial contingency function of task designing, the results of Study 2 empirically verify the dual-path contingency theory via which AI technology adoption promotes employee inventive behavior. Our experimental methodology is limited by intrinsic limitations, even while it allowed for the rigorous manipulation of independent factors and the systematic observation of dependent outcomes, strengthening causal inferences. First, non-probabilistic sampling restricts population generalizability, which may jeopardize external validity even with randomized assignment processes, whereas laboratory testing improves internal validity by controlling the attenuation of confounding variables. Secondly, because of its limited ability to replicate dynamic organizational ecosystems with multilevel interactivity, the controlled experimental environment, despite its scientific benefits, naturally limits ecological validity. This study uses a multi-method triangulation technique, which is in line with accepted methodological paradigms (Li et al., 2015). In particular, the experimental data from Study 2 and the survey-based results from Study 1 cross-validate theoretical claims in a synergistic manner, where behavioral manipulation and psychometric testing work together to support construct operationalization integrity. So the theoretical generalizability and methodological rigor of our findings are significantly improved.

## 8 Discussion

This study presents a dual-process theoretical model that explains how workplace AI technology adoption has paradoxical impacts on employee innovation. It is based on the JD-R paradigm. In particular, we construct two opposing mechanisms: (1) FOCC-mediated activation of cognitive-affective resources (representing AI-enabled cognitive surplus repurposing) and (2) job insecurity-mediated depletion of threat appraisals (resulting from AI-driven occupational identity degradation). Notably, cross-method validation in Studies 1–2 shows that task crafting functions as a differential boundary moderator that bidirectionally regulates these pathways, attenuating negative demand spirals and amplifying the positive resource mobilization effects.

### 8.1 Theoretical implications

This study examines the dichotomous effects of AI technology adoption on employee innovation dynamics and proposes a dual-pathway approach to resolve the organizational paradox (Luo et al., 2022). The two main paths of current scholarly focus are as follows: Early research highlights AI's potential as an empowerment tool, especially through increased cognitive engagement (Zhu et al., 2021) and strategic human capital optimization (Mikalef and Gupta, 2021). Later studies, however, have focused on AI's limiting effects, as shown by the rise in workforce precarity (Liu and Xie, 2024; Wang and Zhou, 2024) and the erosion of normative behavior (Mirbabaie et al., 2022). In order to investigate human-AI congruence effects, a new line of research uses contingency theory. It focuses on how algorithmic complementarity with employee conscientiousness affects task execution efficacy (Man Tang et al., 2022; Du et al., 2022). Our research provides new theoretical insights by systematizing this dichotomy: The AI innovation paradox extends the present nascent understanding of techno-human symbiosis in contexts of digital transformation by appearing as a dialectical occurrence that calls for organizational ambidexterity.

Secondly, using a dual-perspective method that looks at job demands and job resources, this study incorporates the JD-R model to explore the "black box" process connecting the use of AI technology to employee inventive behavior. This builds on earlier studies on the processes by which the use of AI technology affects worker behavior. Using theoretical frameworks including the conservation of resources theory, the cognitive appraisal theory of stress, and self-determination theory, prior research has mostly concentrated on the effects of AI adoption (Zhu et al., 2021; Man Tang et al., 2022; Mirbabaie et al., 2022). Theoretically, this study looks at employment features and suggests that changes brought about by AI technology will unavoidably influence the psychological states and behavioral results of employees (Zhu et al., 2021). In particular, the use of AI technology can have both beneficial and negative effects on employee inventive behavior. The former can provide job resources, such as FOCC, while the latter can increase job demands, such as job instability. The results support the theoretical reasoning that the JD-R model's twin mechanisms of job resources and work demands influence employee inventive behavior when AI technology is applied.

By describing how proactive job sculpting mitigates AI's dialectical innovation effects through dual psychobehavioral pathways, this study operationalizes work control theory in human-AI systems and introduces agentic work redesign as a crucial boundary condition. According to our hypothesis, employees' varied engagement in experiential crafting vs. altering procedural structures results in a range of adaptation patterns to intelligent automation. In particular, our moderated mediation analysis shows that job insecurity and FOCC by AI technology adoption diametrically opposed sensitivity to task crafting magnitude, with high crafting propensity mitigating demand depletion effects and amplifying resource gains.

In order to address recent calls for multilevel analyses of human-AI co-adaptation dynamics, this contingency framework advances three crucial theoretical extensions: (1) establishing task crafting as a dynamic calibration mechanism in technological ambivalence resolution; (2) bridging macro-level work design theory with micro-level proactive behavior literature through techno-agentic interactions; and (3) clarifying the triadic interdependence between AI system characteristics, job architecture fluidity, and employee boundary management competencies.

### 8.2 Practical implications

By establishing demand-regulation safeguards against psycho-behavioral depletion, this study clarifies the paradoxical nature of AI technology adoption in innovation ecosystems and provides organizational leaders with ambidextrous governance frameworks to strategically amplify AI's innovation-enhancing properties.

Firstly, managers want to help their employees see AI technology as a cooperative instrument that improves productivity and adaptability at work rather than as a possible danger to job stability. This manner of framing AI encourages impressions of increased flexibility and less workload, which in turn encourages employee autonomy and initiative in experimenting with new techniques. Managers should also put psychological stability and emotional support first by keeping an eye on workers' emotional states, filling in skill gaps with focused training, and lowering the perceived risks of AI adoption.

Secondly, managers want to stress the importance of encouraging people to create their own tasks. Workers with strong task-crafting inclinations are better able to adjust to AI-driven workplaces, identifying the opportunity for discretion that AI presents while aggressively tackling its drawbacks. As a result, they become more innovative and enthusiastic about their profession. Managers should use personality tests to find applicants who are highly adaptive and proactive, and incorporate AI compatibility and task crafting capabilities into recruitment and selection criteria. In order to guarantee balanced human-machine collaboration, firms should also make investments in skill development and innovation awareness, cultivate an innovative culture, and optimize the allocation of human resources. Managers may overcome the limitations of AI technology while leveraging its potential to stimulate staff innovation by implementing these tactics.

## 9 Conclusion

This study establishes three conceptual breakthroughs to resolve the "AI paradox" in organizational scholarship, it first reframes AI systems within the JD-R framework as dual-valence technological artifacts that exhibit concurrent resource-augmenting and demand-escalating properties. Secondly, it goes beyond conventional models of technology adoption by operationalizing proactive technological adaptivity, which is defined as workers' agentic recalibration of human-AI task interdependencies through goal-oriented job creation. Third, it demonstrates how micro-level agentic actions contingently influence AI's macro-innovation implications by identifying moderation effects, bridging the conceptual ap between techno-optimist and techno-dystopian viewpoints. Three strategic organizational imperatives are derived from the empirical findings:

(1) Strategic AI resource orchestration—Architecting innovation-centric ecosystems through algorithmic job redesign by deploying intelligent systems as cognitive augmentation levers (e.g., automating procedural work to exploratory activities); (2) Proactive identity preservation frameworks—Putting in place psychologically based protections (e.g., AI transparency guidelines and career transition subsidiarity initiatives) to prevent the degradation of one's self-concept brought on by technology displacement;(3) Meta-adaptive capacity cultivation: By instituting dynamic reskilling architectures that prioritize technological stewardship competencies, employees can be transformed from passive recipients of technology to active curators of cyber-physical systems. By minimizing the externalities of technostress and maximizing the innovation yield of anthropo-technological symbiosis, this three-part intervention matrix eventually achieves a strategic balance between workforce sustainability and technology integration.

Three study limitations are acknowledged: (1) The experimental temporal parsimony (Study 2) limits the robustness of causal inference, requiring the adoption of temporally dynamic analytical models to capture hysteresis effects in AI-induced behavioral adaptation; (2) the contextual specificity of our education-sector sample (primarily functional roles) limits ecological validity, requiring cross-industrial validation through comparative studies of AI's innovation impacts across professional archetypes. These initiatives can aid in the investigation of how AI applications in office settings affect the psychology and behavior of workers in this domain.

## Data Availability

The datasets presented in this article are not readily available because several restrictions apply to the dataset used in this study. First, the data collection was limited to full-time employees in technology-intensive industries within China, which may affect the generalizability of findings to other sectors or cultural contexts. Second, the sample consists exclusively of knowledge workers, potentially limiting applicability to manual or service-oriented occupations. Third, the dataset contains only organizational-level variables without individual demographic characteristics due to privacy protection agreements. Researchers interested in accessing the anonymized dataset may contact the corresponding author with a formal request, subject to institutional review board approval and compliance with China's data protection regulations. Requests to access the datasets should be directed to 734378605@qq.com.
